# Aged-associated cytomegalovirus and Epstein-Barr virus reactivation and cytomegalovirus relationship with the frailty syndrome in older women

**DOI:** 10.1371/journal.pone.0180841

**Published:** 2017-07-10

**Authors:** Ronaldo Luis Thomasini, Daniele Sirineu Pereira, Fabiana Souza Máximo Pereira, Elvis Cueva Mateo, Thamires Nader Mota, Gabrielle Gontijo Guimarães, Leani Souza Máximo Pereira, Cristiano Xavier Lima, Mauro Martins Teixeira, Antônio Lúcio Teixeira

**Affiliations:** 1 Faculdade de Medicina de Diamantina (FAMED), NEPii – Núcleo de Estudos de Patologias Infecciosas e Inflamatórias, Universidade Federal dos Vales do Jequinhonha e Mucuri, Diamantina, Minas Gerais, Brazil; 2 Curso de Fisioterapia, Escola de Enfermagem, Universidade Federal de Alfenas, Alfenas, Minas Gerais, Brazil; 3 Faculdade de Medicina, Universidade Federal de Minas Gerais, Belo Horizonte, Minas Gerais, Brazil; 4 Departamento de Fisioterapia, Universidade Federal de Minas Gerais, Belo Horizonte, Minas Gerais, Brazil; 5 Programa de Ciências da Reabilitação, Universidade Federal de Minas Gerais, Belo Horizonte, Minas Gerais, Brazil; 6 Departamento de Fisiologia e Biofísica, Instituto de Ciências Biológicas, Universidade Federal de Minas Gerais, Belo Horizonte, Minas Gerais, Brazil; University of St Andrews, UNITED KINGDOM

## Abstract

Immunosenescence is an age-related reduction of immune system activity that can be associated with frailty. This study aimed to compare cytomegalovirus (CMV) and Epstein–Barr virus (EBV) reactivations (based on viremias) between young and elderly women who had a chronic CMV and/or EBV infection (i.e., an IgG^+^ serostatus) without an acute infection (i.e., an IgM^−^ serostatus), and among the elderly group categorized according to frailty status. DNA was extracted from plasma using standard protocols and serostatus was determined by enzyme-linked immunosorbent assay. Quantitative real-time polymerase chain reaction analyses for CMV and EBV were carried out and viral loads were determined. Among elderly women (*n* = 71), 59% were positive for CMV, in contrast to only 8% of young women (*n* = 73). Elderly women classified as frail, pre-frail, and non-frail presented 82%, 56%, and 48% positivity for CMV, respectively. Frequency and viral load were significantly higher in the elderly group vs. the young group (*p* < 0.0001 and *p* = 0.01, respectively) and in elderly with frailty vs. those without frailty (*p* = 0.007 and *p* = 0.03, respectively). The frequency of CMV reactivation presented odds ratios of 11.77 for aging and 6.13 for frailty, and relative risks of 5.39 for aging and 1.93 for frailty. EBV was detected in 30% of the elderly women and 15% of the young women (*p* = 0.04); however, the viral load did not significantly differ between the two age groups. The frequency of EBV reactivation presented odds ratios of 2.36 for aging and 2.90 for frailty, and relative risks of 1.96 for aging and 2.12 for frailty. However, no difference in EBV viral load among the frailty status subgroups was found. In conclusion, the frequency of CMV reactivation was associated with aging and ongoing frailty, whereas the frequency of EBV reactivation was associated only with aging.

## Introduction

The process of aging is accompanied by chronic diseases that can be controlled without impairing the independence and autonomy of the individual. However, a significant portion of the elderly population has conditions, constituting ‘frailty’, that render them more vulnerable to harm and increase their risk of experiencing adverse events. The term frailty has been used frequently in the literature, but its precise definition has evolved during recent years. According to Fried [1], frailty is a state of increased vulnerability that is associated with aging and results from a reduced physiological reserve and a decreased ability to maintain homeostasis under stress. It is a multi-systemic and highly prevalent syndrome that affects elderly individuals and can result in serious morbidity and mortality [2,3].

Three factors may influence frailty: (1) dysfunction of the neuroendocrine system, (2) dysfunction of the immune system (immunosenescence) [4], and (3) sarcopenia [5]. Alterations in the neuroendocrine and immune systems have been shown to influence muscular and functional changes in older adults [6]. In Brazil, the risk of frailty increases with age and occurs in 10–25% of people aged over 65 years and 46% of those aged over 85 years [7,8].

There are many measures of frailty, but the phenotypic model proposed by Fried [1] is the most widely used in research and clinical practice. It includes: *weight loss* (self-reported weight loss of more than 4.5 kg or recorded weight loss of ≥ 5% per year); *self-reported exhaustion* (self-reported exhaustion for 3–4 days per week or most of the time based on the US Center for Epidemiological Studies Depression Scale); *low energy expenditure* (energy expenditure < 383 kcal/week for men or < 270 kcal/week for women); *slow gait speed* (standardized cutoff times to walk 4.57 m, stratified by sex and height); and *weak grip strength* (grip strength, stratified by sex and body-mass index [1]). This model classifies the elderly as non-frail (meeting no criteria of frailty from the above metrics), pre-frail (meeting 1 or 2 criteria), and frail (meeting 3 or more criteria) [5,9].

Immunosenescence is among the three most important factors that affect frailty [4]. Immunosenescence is characterized by age-related dysfunction of the immune system and is associated with an increased frequency of vaccination inefficacy, infections, and auto-reactivity [10]. Elderly individuals may also present low-grade chronic inflammation, even in the absence of comorbidities, which is described in the literature as 'inflammaging' and defined as chronically elevated levels of pro-inflammatory cytokines [3,11].

Recently, several herpesviruses have been studied regarding their possible associations with immunosenescence [12–14]. Cytomegalovirus (CMV) and Epstein–Barr virus (EBV) are members of the *Herpesviridae* family that can establish latent infections in a host. The reactivation of latent CMV or EBV infection can occur after the use of immunosuppressive agents or in individuals who have developed acquired immune deficiency syndrome [15]. However, severe immunosuppression does not seem to be strictly necessary for herpesviral reactivation to occur. For instance, labial lesions caused by recurrent herpes simplex virus may occur in immunocompetent individuals after exposure to cold, sunlight, lip injury, and stress [16]. The frequent shedding of viable EBV and human herpesvirus (HHV)-7 particles in saliva, probably as a result of replication in salivary glands, also supports the notion that other herpesviruses may undergo reactivation at low levels in immunocompetent individuals [17].

Herpesviral reactivation can be asymptomatic, especially in cases with a low viremia. Herpesvirus latency results from a balance between minimal viral replication, the activity of specific CD8^+^ T cells, and stable circulating concentrations of antibodies against the virus [18–20]. Furthermore, age-related declines in cellular immunity can be associated with increased viral replication. Although aging-associated herpesvirus reactivation has been reported in the literature, the clinical impact of viral replication and its potential link with frailty in the elderly remain to be explored.

The aim of this study was to compare CMV and/or EBV reactivations (based on viremias) in elderly and young women who had both an IgG^+^ serostatus (i.e., chronic CMV/EBV infection) and an IgM^−^ serostatus (i.e., no acute CMV/EBV infection). We also evaluated the presence and levels of viremia in elderly people categorized into three frailty subgroups.

## Materials and methods

### Control group

Healthy young women (18–30 years old, undergraduate or post-graduate students) were recruited. Individuals who described any signs, symptoms, or a recent history of acute infections were excluded because of possible primary CMV or EBV infections.

### Elderly group

The group was composed of women (60–80 years old) who participated in a previous cross-sectional study of frailty conducted in the Department of Physiotherapy, Federal University of Minas Gerais, Brazil [21]. The elderly women were classified into three subgroups (frail, pre-frail, and non-frail) according to the scheme proposed by Fried [1]. Briefly, frailty was defined as a condition meeting three of the following five phenotypic criteria: *weight loss* (self-reported weight loss of more than 4.5 kg or recorded weight loss of ≥ 5% per year); *self-reported exhaustion* (self-reported exhaustion for 3–4 days per week or most of the time based on the US Center for Epidemiological Studies Depression Scale); *low energy expenditure* (energy expenditure < 383 kcal/week for men or < 270 kcal/week for women); *slow gait speed* (standardized cutoff times to walk 4.57 m, stratified by sex and height); and *weak grip strength* (grip strength, stratified by sex and body-mass index), which is associated with an increased risk for poor health outcomes, including falls, incident disability, hospitalization, and mortality [1,22].

Only women were included in this study because fewer male individuals attended the department where this study was conducted. In addition, a ‘feminization phenomenon’ of the aged Brazilian population has previously been reported to have occurred owing to gender differences in mean longevity. The control group also included only women to minimize bias related to gender.

### CMV and EBV serostatuses

Individuals from the control and elderly groups who were CMV or EBV IgM^+^ were excluded from the study because we intended to investigate chronically infected cases undergoing reactivation, rather than acute viral infection. Individuals who were CMV and EBV IgG^−^ were also excluded because natural viral reactivation cannot occur in IgG seronegative subjects.

### Sample collection

Blood was collected by venipuncture using a commercial vacuum system with ethylenediaminetetraacetic acid (EDTA) anticoagulant (Vacutainer^®^, BD Biosciences, Franklin Lakes, NJ, USA). Plasma was separated immediately after centrifugation and then frozen at −70°C. To avoid repeated freezing and thawing, plasma was divided into aliquots in sterile plastic micro tubes.

### DNA extraction

Before DNA extraction, 50,000 copies (per milliliter of plasma) of a linearized plasmid containing a specific exogenous DNA (equine herpesvirus 1) were added to each plasma sample as an internal control for the quality of DNA extraction and the presence of polymerase chain reaction (PCR) inhibitors. Sample (200 μL of plasma) was incubated for 2 h at 56°C with 100 μL lysis buffer (10 mM Tris-HCl [pH 8.3], 150 mM NaCl, 1 mM EDTA, and 0.2% sodium dodecyl sulfate) and 5 μL of 20 mg/mL proteinase K. After incubation, 100 μL of phenol-chloroform-isoamylic alcohol (25:24:1) was added and then centrifuged at 12,000 rpm for 5 min. The supernatant was transferred to another tube and the last step was repeated twice more. DNA was precipitated with 3 volumes of isopropanol and 20 μL of 3 M sodium acetate (pH 5.2). The sedimented DNA was washed twice by centrifugation with 500 μL of 70% ethanol, dried at room temperature, rehydrated with 20 μl of Tris-EDTA buffer, and stored at −70°C until analysis.

### Ethical concerns

This study was approved by the Research Ethics Institutional Committee of the Federal University of Minas Gerais (UFMG) and conducted in compliance with the Helsinki Declaration. In addition, all participants provided written informed consent before participating in this study.

### Enzyme-linked immunosorbent assays (ELISAs) for CMV and EBV

ELISAs for antibodies to CMV and EBV viral capsid antigen (VCA) IgG and IgM (Serion^®^, Wurzburg, DEU) were carried out to determine each participant’s serostatus following the manufacturer’s instructions.

### CMV and EBV gene cloning and plasmid construction

Standards consisted of serial dilutions of plasmids containing inserted CMV or EBV DNA segments. Briefly, PCR amplicons of CMV (gB) or EBV (EBER) were cloned into TOPO^®^ plasmids using a topoisomerase kit (PCR^®^2.1 TOPO^®^, Life Technologies., Carlsbad, CA, USA). *Escherichia coli* (*E*. *coli*) XL1 Blue was transformed with these plasmids and positive clones were selected by a blue-white screening protocol using isopropyl β-ᴅ-1-thiogalactopyranoside/5-bromo-4-chloro-3-indolyl-β-ᴅ-galactopyranoside plates and ampicillin. The selected *E*. *coli* clones were proliferated in Luria–Bertani broth, their plasmid DNA was extracted, and the presence of the DNA inserts was confirmed by conventional PCR.

Plasmid DNA was linearized with the restriction enzyme EcoRV (Anza^™^, Life Technologies) and its concentration was estimated by a fluorometric assay using a Qubit^™^ 2.0 fluorometer (Thermo Fisher Scientific, Waltham, MA, USA). Serial dilutions were carried out to obtain standards containing 10^6^, 10^5^, 10^4^, 10^3^, 10^2^, and 10 copies of DNA target per microliter for each virus.

### Definitions

Positivity for CMV or EBV (viremia) was defined as a single positive quantitative PCR (qPCR) result for DNA extracted from the plasma considering all the quality criteria described below. Viral reactivation was defined based on viremia associated with IgG^+^/IgM^−^ serostatus for each virus.

### Detection of CMV and EBV by qPCR

The same qPCR protocol was used for both viruses, except with different primers and standards. Reactions were carried out using the SYBR^®^ Green detection protocol and StepOne^™^ real-time PCR equipment (Life Technologies). Each PCR mixture consisted of 900 nM of each primer for CMV (forward: 5′-CGACGAAACGTCAAAACCTT-3′ and reverse: 5′-TACCCCTATCGCGTGTGTTC-3′; CMV glycoprotein B, GenBank accession: U66425.1, positions 2273–2292 and 2441–2460) or 900 nM of each primer for EBV (forward: 5′-CCCGCCTACACACCAACTAT-3′ and reverse: 5′-AGTCTGGGAAGACAACCACA-3′; EBV EBER 1 and 2, GenBank accession: J02078.1, positions 81–100 and 271–290), 2.5 μL of DNA samples or standard, 5 μL of SYBR^®^ Green master mix (master mix real-time reagent), and nuclease-free water in a total reaction volume of 10 μL. Each reaction was carried out in a 48-well plate with 2 blank reactions, 6 standards (as described above), and samples.

An additional qPCR analysis was performed for the exogenous plasmid DNA added before DNA extraction that was used as an internal quality control. DNA samples that presented < 80% extraction efficiency (of the initial 50,000 plasmid copies) were discarded and DNA extraction was repeated.

The conditions of the reaction were: initial denaturation and enzyme activation for 10 min at 94°C, followed by 40 cycles of 15 s at 94°C for denaturation, 20 s at 56°C for primer annealing, and 72°C for 1 min for extension and data collection. Finally, a melting curve analysis was carried out and data were analyzed using the software packaged with the thermal cycler to validate the reactions and calculate the number of viral copies per reaction.

The number of viral copies per milliliter of plasma (viral load) was calculated considering the volume of sample used, the efficiency of the DNA extraction, the volume of water used to rehydrate DNA, and the volume of DNA used in the PCR reaction.

### Specificity and sensitivity tests

Clinical samples showing positivity for CMV and EBV samples positive for another five human herpesviruses (herpes simplex virus, varicella zoster virus, HHV-6, HHV-7, and HHV-8), and negative samples were tested by qPCR following the techniques described above to evaluate the specificity of qPCR. Several dilutions of the plasmids constructed for CMV and EBV were also tested by the same technique. The sensitivity of qPCR was defined as the lowest amount of the DNA target that yielded positive results for 10 replicates. A strain that is used internationally as a standard for the nucleic acid testing of CMV (CMV Merlin strain, lot number 09/162) was used to evaluate the calibration curve of qPCR. The lower limit of detection was 400 copies/mL and the detection curve was linear up to 10^7^ copies/mL for both viruses.

### Statistical analysis

Comparison among groups was performed by chi-squared, Kruskal–Wallis, and Mann–Whitney U tests using Prism Graph Pad 5.0 (Graph Pad Software Inc., San Diego, CA, USA). *p*-Values < 0.05 were considered statistically significant. In addition, the odds ratio, relative risk, sensitivity, and specificity were calculated to evaluate whether the presence of viruses was associated with aging or frailty status.

## Results

The qPCR assays for CMV and EBV both yielded standard curves with *R*^*2*^ = 0.99. Neither nonspecific amplification nor cross-reaction with other human herpesviruses was found, and the lower bound of sensitivity was 400 viral copies/mL. In the young group (*n* = 79), 73 individuals (92.4%) were CMV IgG^+^/IgM^−^ and EBV IgG^+^/IgM^−^. Among the remaining individuals, four subjects (5%) were CMV IgG^−^/EBV IgG^−^, one (1.2%) was CMV IgG^+^/EBV IgG^−^, and another (1.2%) was CMV IgG^−^/EBV IgG^+^. No participants were IgM^+^. In the elderly group (total *n* = 75; non-frail *n* = 27; pre-frail *n* = 24; and frail *n* = 24), 71 individuals (94.6%) were CMV IgG^+^/IgM^−^ and EBV IgG^+^/IgM^−^, three individuals (4%) were CMV IgG^−^/EBV IgG^−^, and only one individual (1.3%) was CMV IgM^+^. Differences in serostatuses between groups and subgroups were not statistically significant.

The individual who was CMV IgM^+^ was excluded to avoid confusion related to an acute infection. Individuals without specific IgG against one or both viruses (6 individuals from the young group and 3 individuals from the elderly group) were considered to not have been previously infected and therefore viral reactivation could not occur; thus, those individuals were excluded from the study. Based on ELISA for antibodies to CMV and EBV (IgG and IgM) results and frailty status, 71 elderly women and 73 young women were selected for qPCR testing of their samples. The 71 elderly women were classified as non-frail (*n* = 26, 36.6%), pre-frail (*n* = 23, 32.4%), and -frail (*n* = 22, 31.0%).

Among the elderly women, 42/71 (59%) presented CMV DNA in their plasma (viremia indicating CMV reactivation), in contrast with only 8/73 (10%) of the young women (*p* < 0.0001). Elderly women classified as frail, pre-frail, and non-frail had CMV DNA in 18/22 (82%), 13/23 (56%), and 11/26 (42%) of cases, respectively. CMV DNA was detected significantly more frequently in frail elderly than in other elderly subgroups (*p* = 0.007).

**Fig 1:**
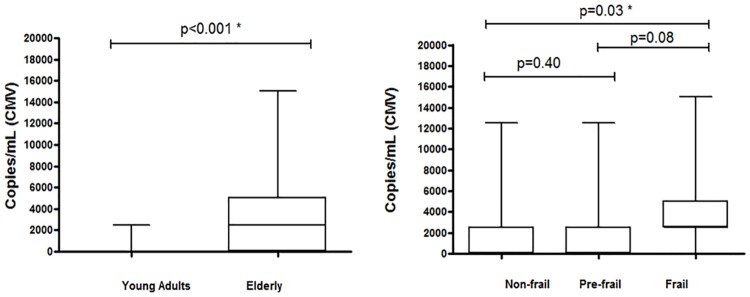
(a) Distribution of cytomegalovirus (CMV) genome copy numbers (viral load) in samples obtained from the elderly and young groups. (b) Distribution of CMV genome copy numbers (viral load) in samples obtained from elderly classified into three subgroups: non-frail, pre-frail, and frail.

The average CMV viral load in the elderly group was 2,887 copies/mL (standard deviation [SD]: 3,675; range: 0–15,000), whereas it was 273 copies/mL (SD: 786; range: 0–2,500) in the young group. The viral load was significantly higher in the elderly group than the young group (*p* < 0.001), and higher in the frail elderly subgroup than in other subgroups (*p* = 0.03) (Fig 1a and 1b).

EBV DNA in plasma (viremia indication EBV reactivation) was found in 21/71 (30%) of elderly women and in 11/73 (15%) of young women (*p* = 0.04). Elderly classified as frail, pre-frail, and non-frail had EBV DNA in 9/22 (41%), 7/23 (30%), and 5/26 (19%) of cases, respectively (*p* = non-significant [NS]).

**Fig 2:**
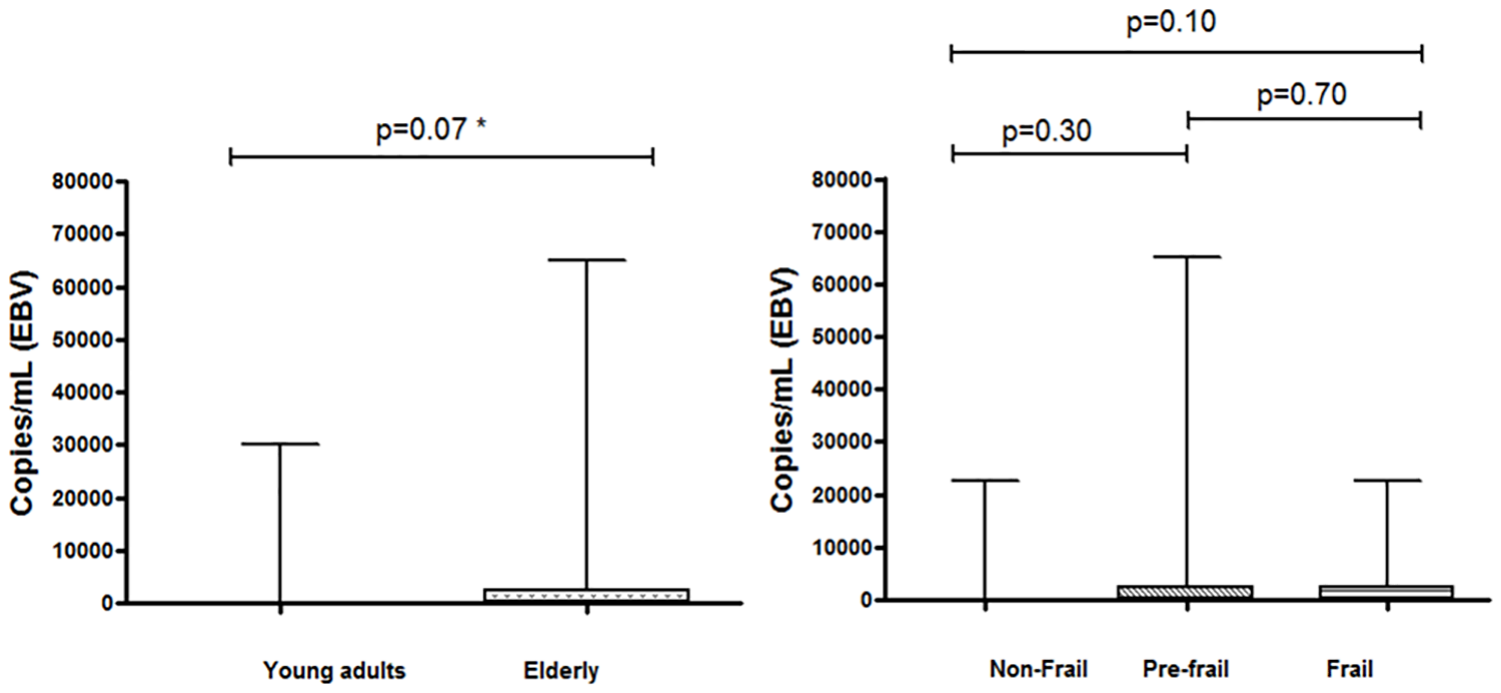
(a) Distribution of Epstein–Barr virus (EBV) genome copy numbers (viral load) in samples obtained from the elderly and young groups. (b) Distribution of EBV genome copy numbers (viral load) in samples obtained from elderly women classified into three subgroups: non-frail, pre-frail, and frail.

The average EBV viral load in the elderly group was 2,711 copies/mL (SD: 8,780; range: 0–65,000), whereas in the young group it was 1,472 copies/mL (SD: 4,523; range: 0–30,000) (*p* = NS; Fig 2a). No significant differences were found with respect to EBV viral loads between the frailty status subgroups of the elderly group (Fig 2b).

Among individuals who were positive for at least one virus as deduced by the qPCR results, CMV and EBV were detected simultaneously (co-reactivation) in 42%, 42%, and 23% of frail, pre-frail, and non-frail elderly, respectively (*p* = NS). Table 1 shows the odds ratio, relative risk, sensitivity, and specificity for frailty, pre-frailty, and aging (independently of frailty) of both viruses.

**Table 1:** CMV and EBV DNA in plasma (viremias indicating reactivations) compared with aging and frailty status.

	CMV *positive*			EBV *positive*		
	Aging	Pre-Frailty	Frailty	Aging	Pre-Frailty	Frailty
Odds ratio	11.77	1.77	6.13	2.36	1.83	2.90
Relative risk	5.39	1.33	1.93	1.96	1.58	2.12
Sensitivity	84%	54%	62%	65%	58%	64%
Specificity	69%	60%	79%	55%	56%	61%
*P*-value	<0.0001*	0.39	0.007*	0.04*	0.50	0.12

* Chi-squared test.

## Discussion

Most herpesviruses are ubiquitous and persist in a latent state in their hosts for life. However, some viruses in this family, such as human herpes simplex virus 2 and human herpesvirus 8 (HHV-8 or Kaposi's sarcoma-associated herpesvirus), are associated with lower seroprevalences than other herpesviruses. Seroprevalence depends on the type of herpesvirus and the age of a host, and can vary geographically. The infection can become reactivated after immunosuppressive therapy and other conditions that affect immune function [15,23]. The clinical outcome of viral reactivation depends on the type of disease, severity of immunosuppression, and clinical baseline of the subjects. However, severe immunosuppression is not a *sine qua non* condition for herpesviral reactivation, and viral reactivation can be asymptomatic, especially in cases with low viral loads [17,24,25].

In this study, we reported a higher frequency and greater viral load of CMV in elderly women then in young women. In addition, an association between CMV and frailty was found, i.e., frail elderly individuals were more likely to exhibit viral reactivation and to have higher viral loads. Whereas age and frailty correlated with the frequency of reactivation and the viral load in subjects with CMV, age correlated with the frequency of reactivation but not the viral load in subjects with EBV. In addition, EBV was not associated with frailty, but there was increased risk of frailty in older women who were positive for CMV.

Other authors have reported associations between CMV and aging or frailty [26–28]. The present study broadly corroborates those previous reports, but differs from them in some respects. Most studies correlated IgG antibodies titers to CMV with the grade of immunosenescence, which is an indirect method to estimate viral replication. However, testing of paired samples is necessary to determine a significant rise in IgG titer. Therefore, a higher level of IgG antibodies against CMV does not indicate *per se* that reactivation has occurred [29].

In this study, we used qPCR, which is a direct method for assessing the presence of the virus, and only individuals who were positive for IgG antibodies to CMV and/or EBV were included, since no viral reactivation can occur in individuals who are not latently infected. Indeed, if CMV reactivation based on viremia is used as a biomarker for immunosenescence, false negative results can occur when a person does not have a previous latent CMV infection. Thus, previous serological testing or detection of viral DNA (latent state) in leukocytes is important for correctly interpreting the results of molecular methods.

Our testing method did not show a high sensitivity and specificity, but presented significant odds ratios and relative risk factors for aging and frailty. Naturally, cut-off values remain to be determined based on studies in more subjects, but a higher level of viral DNA indicates the need for the careful follow up of a subject. The association of qPCR with IgG antibodies titers to CMV could suggest that qPCR may be applicable as an alternative protocol for evaluating immunosenescence and frailty risk. Furthermore, the sensitivity and specificity of viral detection may be enhanced when the results of serological and molecular tests are interpreted together.

We agree with the need to further test the hypothesis that CMV reactivation indirectly contributes to frailty. CMV-infected cells can experience complex changes, leading to the up or down regulation of factors that may contribute to the pathogenesis of inflammatory processes such as ‘inflammaging’. CMV can infect several types of cells (T lymphocytes, monocytes/macrophages, dendritic cells, epithelial cells, fibroblasts, endothelial cells, and muscle cells); therefore, many tissues can be affected by a systemic CMV infection [30]. CMV can also suppress the function of antigen-specific T cytotoxic cells [31]. Immunosuppressive effects of CMV that directly impair the functioning of lymphocytes and induce the production of auto-antibodies and inflammatory mediators have been described [30].

Hypothetically, a ‘two-way’ mechanism could contribute to the weak control of viral replication: CD8^+^ T cells fail to monitor infected cells due to senescence, and viral replication leads to a decrease in cell-mediated immune function. This suggests an age-related mechanism in which immunosenescence and the virus can act in synergy to facilitate viral immune evasion.

Sequential events that begin with the release of cytokines and chemokines from virus-infected cells can orchestrate the activation of immune cells and lead to inflammation. Another potential impact of viral infection is an impairment of immune functions due to viral replication, which may facilitate simultaneous infection by other agents [32].

Alternatively, viral replication associated with an increase in anti-CMV IgG titers could merely indicate a failure of the immune system to control the latent infection. Associations have been reported between CMV and numerous types of illness and disease. Thus, a critical evaluation of each report, and especially the methods of detection used, is necessary. Qualitative PCR performed with DNA extracted from leukocytes can yield false positive results because of the presence of viral DNA within cells harboring the virus [33,34]. Despite this, we suggest that CMV load could be considered as a biomarker for frailty risk in elderly individuals who are latently infected with CMV.

Another point that remains to be discussed is whether a laboratory biomarker really helps in clinical decisions. Is an intervention possible? Is CMV vaccination, similar to varicella-zoster vaccination, indicated (when available in the future)? Would the patients benefit from anti-CMV therapy? Although the proportion of CMV positivity in the elderly group was higher, some individuals in the young group were positive for CMV. Although an absence of viral DNA in the plasma of individuals latently infected is theoretically possible, a few DNA copies can usually be detected because of the lysis of cells that harbor virus. The high sensitivity of real-time PCR allows the detection of low viral loads in the latent stage of infection, and a positive test does not always indicate ‘true’ viral reactivation [34]. Therefore, it may be important to define appropriate cut-off values.

We found that EBV, in contrast to CMV, was associated with aging but not with frailty. Some previous reports also describe an association between EBV antibody titers and aging [35,36]. We cannot discard the possibility that our results are biased, but the qPCR method used in this study has been exhaustively tested. Furthermore, an internal control for the quality of extracted DNA and the presence of PCR inhibitors was included in testing, along with clinical samples and several plasmid dilutions. Thus, at least in this cohort, EBV did not appear to be associated with frailty. While CMV is harbored by T cells, EBV is harbored by B cells, and EBV may not act as an antigen stressor in the way that CMV does.

Latent EBV infection in B cells is controlled by natural killer cells and CD4^+^ and CD8^+^ T cells, which prevent EBV-induced B cell proliferation. Various malignancies have been associated with EBV infection, including nasopharyngeal carcinoma, lymphomas (such as Burkitt’s, Hodgkin’s, T cell non-Hodgkin’s lymphoma, and human immunodeficiency virus-related non-Hodgkin’s lymphoma), and post-transplant lymphoproliferative disorder [37]. The largest clinical impact of immunosenescence in EBV-infected subjects is an increased risk of lymphoproliferative disorders.

The association of EBV with aging, but not frailty, reinforces the idea that EBV and CMV play different roles in immunosenescence. The presence of EBV DNA in plasma could be strictly related to a failure of CD8^+^ T cells to control the infection, whereas the presence of CMV could have effects such as an increased risk of frailty. In this study, associations between CMV and both aging and frailty, and between EBV and aging, were identified using a direct method to detect the presence of the viruses in young and elderly women. The results differ from those of other studies that used indirect methods such as antibody levels to infer viral reactivation. The association of frailty with CMV load is a new contribution of this study, as well as the proposal of a ‘two-way’ hypothesis to explain viral immune evasion. The mechanisms of cellular injury by CMV and EBV remain to be further explored.

In conclusion, this study demonstrates, by virus-specific DNA detection, a clear association between viral reactivation and aging, as well as the risk of frailty, in elderly women.

## Supporting information

S1 Table Supplementary data (underlying of findings). Status of frailty of the studied cohort, qPCR results for CMV and EBV, and serology IgG/IgM for CMV and EBV.
(PDF)
