# Supplementary material for: Aged-associated cytomegalovirus and Epstein-Barr virus reactivation and cytomegalovirus relationship with the frailty syndrome in older women
Source: PLoS One. 2017 Jul 10;12(7):e0180841. doi: 10.1371/journal.pone.0180841 (PMC5507269; doi:10.1371/journal.pone.0180841)
Supplement: S1 Table — Status of frailty of the studied cohort, qPCR results for CMV and EBV, and serology IgG/IgM for CMV and EBV. (PDF) [file pone.0180841.s001.pdf]

Supplementary data (underlying of findings).

Sample Id	Group	Frailty Status	qPCR CMV (copies/mL)	qPCR EBV (copies/mL)	ELISA CMV IgG	ELISA EBV IgG	ELISA CMV IgM	ELISA EBV IgM
94	Elderly	Frail	2500	0	+	+	-	-
132	Elderly	Frail	15000	0	+	+	-	-
174	Elderly	Frail	0	2500	+	+	-	-
207	Elderly	Frail	5000	2500	+	+	-	-
209	Elderly	Frail	2500	0	+	+	-	-
237	Elderly	Frail	12500	0	+	+	-	-
250	Elderly	Frail	5000	0	+	+	-	-
255	Elderly	Frail	7500	0	+	+	-	-
288	Elderly	Frail	2500	2500	+	+	-	-
296	Elderly	Frail	5000	2500	+	+	-	-
336	Elderly	Frail	2500	2500	+	+	-	-
351	Elderly	Frail	2500	7500	+	+	-	-
370	Elderly	Frail	0	0	+	+	-	-
375	Elderly	Frail	5000	0	+	+	-	-
376	Elderly	Frail	2500	22500	+	+	-	-
387	Elderly	Frail	0	0	+	+	-	-
398	Elderly	Frail	0	0	+	+	-	-
400	Elderly	Frail	2500	0	+	+	-	-
406	Elderly	Frail	2500	0	+	+	-	-
426	Elderly	Frail	12500	2500	+	+	-	-
462	Elderly	Frail	2500	0	+	+	-	-
470	Elderly	Frail	2500	2500	+	+	-	-
188*	Elderly	Frail	ND	ND	+	+	+	-
201*	Elderly	Frail	ND	ND	-	-	-	-
106	Elderly	Pre-frail	0	0	+	+	-	-
133	Elderly	Pre-frail	0	0	+	+	-	-
165	Elderly	Pre-frail	0	0	+	+	-	-
166	Elderly	Pre-frail	0	0	+	+	-	-
187	Elderly	Pre-frail	2500	0	+	+	-	-
204	Elderly	Pre-frail	0	0	+	+	-	-
228	Elderly	Pre-frail	2500	2500	+	+	-	-
252	Elderly	Pre-frail	7500	0	+	+	-	-
278	Elderly	Pre-frail	2500	12500	+	+	-	-
280	Elderly	Pre-frail	10000	0	+	+	-	-
287	Elderly	Pre-frail	12500	0	+	+	-	-
312	Elderly	Pre-frail	5000	65000	+	+	-	-
317	Elderly	Pre-frail	2500	2500	+	+	-	-
320	Elderly	Pre-frail	2500	0	+	+	-	-
350	Elderly	Pre-frail	0	0	+	+	-	-
361	Elderly	Pre-frail	5000	12500	+	+	-	-
402	Elderly	Pre-frail	0	0	+	+	-	-

Supplementary data (Continued).

403	Elderly	Pre-frail	0	0	+	+	-	-
407	Elderly	Pre-frail	2500	15000	+	+	-	-
416	Elderly	Pre-frail	2500	0	+	+	-	-
418	Elderly	Pre-frail	2500	0	+	+	-	-
442	Elderly	Pre-frail	0	0	+	+	-	-
453	Elderly	Pre-frail	0	2500	+	+	-	-
283*	Elderly	Pre-frail	ND	ND	-	-	-	-
93	Elderly	Non frail	2500	0	+	+	-	-
97	Elderly	Non frail	0	0	+	+	-	-
102	Elderly	Non frail	5000	22500	+	+	-	-
103	Elderly	Non frail	0	0	+	+	-	-
108	Elderly	Non frail	0	0	+	+	-	-
115	Elderly	Non frail	0	0	+	+	-	-
124	Elderly	Non frail	0	0	+	+	-	-
127	Elderly	Non frail	2500	0	+	+	-	-
135	Elderly	Non frail	0	0	+	+	-	-
160	Elderly	Non frail	0	0	+	+	-	-
162	Elderly	Non frail	0	0	+	+	-	-
163	Elderly	Non frail	5000	0	+	+	-	-
163	Elderly	Non frail	0	2500	+	+	-	-
171	Elderly	Non frail	2500	2500	+	+	-	-
196	Elderly	Non frail	0	0	+	+	-	-
213	Elderly	Non frail	0	0	+	+	-	-
215	Elderly	Non frail	2500	2500	+	+	-	-
218	Elderly	Non frail	5000	0	+	+	-	-
221	Elderly	Non frail	0	2500	+	+	-	-
239	Elderly	Non frail	2500	0	+	+	-	-
240	Elderly	Non frail	0	0	+	+	-	-
271	Elderly	Non frail	0	0	+	+	-	-
275	Elderly	Non frail	2500	0	+	+	-	-
275	Elderly	Non frail	0	0	+	+	-	-
285	Elderly	Non frail	10000	0	+	+	-	-
290	Elderly	Non frail	12500	0	+	+	-	-
98*	Elderly	Non frail	ND	ND	-	-	-	-
C1	Control	NA	0	0	+	+	-	-
C2	Control	NA	0	0	+	+	-	-
C3	Control	NA	0	0	+	+	-	-
C4*	Control	NA	ND	ND	-	-	-	-
C5*	Control	NA	ND	ND	-	-	-	-
C6	Control	NA	2500	0	+	+	-	-
C7	Control	NA	0	0	+	+	-	-
C8	Control	NA	0	0	+	+	-	-
C9	Control	NA	0	0	+	+	-	-
C10	Control	NA	0	0	+	+	-	-
C11	Control	NA	0	0	+	+	-	-

Supplementary data (Continued).

C12	Control	NA	0	0	+	+	-	-
C13	Control	NA	0	0	+	+	-	-
C14	Control	NA	0	0	+	+	-	-
C15	Control	NA	0	0	+	+	-	-
C16	Control	NA	0	10000	+	+	-	-
C17*	Control	NA	ND	ND	-	-	-	-
C18	Control	NA	0	0	+	+	-	-
C19	Control	NA	0	0	+	+	-	-
C20	Control	NA	0	0	+	+	-	-
C21	Control	NA	0	0	+	+	-	-
C22*	Control	NA	ND	ND	-	-	-	-
C23	Control	NA	0	0	+	+	-	-
C24	Control	NA	2500	17500	+	+	-	-
C25	Control	NA	0	0	+	+	-	-
C26	Control	NA	2500	7500	+	+	-	-
C27	Control	NA	0	0	+	+	-	-
C28*	Control	NA	ND	ND	+	-	-	-
C29	Control	NA	0	0	+	+	-	-
C30	Control	NA	0	0	+	+	-	-
C31	Control	NA	0	0	+	+	-	-
C32	Control	NA	0	0	+	+	-	-
C33	Control	NA	0	0	+	+	-	-
C34	Control	NA	0	0	+	+	-	-
C35	Control	NA	0	0	+	+	-	-
C36	Control	NA	0	0	+	+	-	-
C37	Control	NA	0	0	+	+	-	-
C38	Control	NA	0	0	+	+	-	-
C39	Control	NA	0	0	+	+	-	-
C40	Control	NA	0	0	+	+	-	-
C41	Control	NA	0	5000	+	+	-	-
C42	Control	NA	0	0	+	+	-	-
C43	Control	NA	0	0	+	+	-	-
C44	Control	NA	0	0	+	+	-	-
C45	Control	NA	0	5000	+	+	-	-
C46	Control	NA	0	0	+	+	-	-
C47	Control	NA	0	0	+	+	-	-
C48	Control	NA	0	0	+	+	-	-
C49	Control	NA	0	0	+	+	-	-
C50	Control	NA	2500	0	+	+	-	-
C51	Control	NA	0	0	+	+	-	-
C52	Control	NA	0	0	+	+	-	-
C53	Control	NA	0	0	+	+	-	-
C54	Control	NA	0	0	+	+	-	-
C55	Control	NA	0	0	+	+	-	-
C56	Control	NA	0	0	+	+	-	-

Supplementary data (Continued).

C57	Control	NA	2500	0	+	+	-	-
C58	Control	NA	0	0	+	+	-	-
C59	Control	NA	0	7500	+	+	-	-
C60	Control	NA	0	5000	+	+	-	-
C61	Control	NA	0	0	+	+	-	-
C62	Control	NA	0	0	+	+	-	-
C63	Control	NA	0	0	+	+	-	-
C64	Control	NA	0	0	+	+	-	-
C65	Control	NA	0	0	+	+	-	-
C66*	Control	NA	ND	ND	-	+	-	-
C67	Control	NA	2500	0	+	+	-	-
C68	Control	NA	0	0	+	+	-	-
C69	Control	NA	0	7500	+	+	-	-
C70	Control	NA	0	5000	+	+	-	-
C71	Control	NA	0	0	+	+	-	-
C72	Control	NA	0	0	+	+	-	-
C73	Control	NA	0	0	+	+	-	-
C74	Control	NA	0	0	+	+	-	-
C75	Control	NA	0	0	+	+	-	-
C76	Control	NA	0	0	+	+	-	-
C77	Control	NA	2500	7500	+	+	-	-
C78	Control	NA	2500	30000	+	+	-	-
C79	Control	NA	0	0	+	+	-	-

ND: Not done.
NA: Not applicable.
***** Excluded from cohort (Please, see ‘Material and Methods’ for more details).
